# A-769662 Protects Osteoblasts from Hydrogen Dioxide-Induced Apoptosis through Activating of AMP-Activated Protein Kinase (AMPK)

**DOI:** 10.3390/ijms150611190

**Published:** 2014-06-23

**Authors:** Yalong Zhu, Jianhua Zhou, Rongguang Ao, Baoqing Yu

**Affiliations:** Orthopedics Department, Shanghai Pudong Hospital, Fudan University Pudong Medical Center, Pudong, Shanghai 201399, China; pdzhuzhu@hotmail.com (Y.Z.); jianhuazhou126@126.com (J.Z.); doctorybq@163.com (R.A.)

**Keywords:** osteonecrosis, AMPK, A-769662, oxidative stress and apoptosis

## Abstract

Here we report that 5'-monophosphate (AMP)-activated protein kinase (AMPK) agonist A-769662 inhibited hydrogen peroxide (H_2_O_2_)-induced viability loss and apoptosis of human and mouse osteoblast cells. H_2_O_2_-induced moderate AMPK activation in osteoblast cells, which was enhanced by A-769662. Inactivation of AMPK by its inhibitor compound C, or by target shRNA-mediated silencing and kinase dead (KD) mutation exacerbated H_2_O_2_-induced cytotoxicity in osteoblast cells. A-769662-mediated protective effect against H_2_O_2_ was also blocked by AMPK inhibition or depletion. A-769662 inhibited reactive oxygen species (ROS) accumulation by H_2_O_2_ in osteoblast cells. Meanwhile, H_2_O_2_-induced ATP depletion was inhibited by A-769662, but was aggravated by compound C. Further, H_2_O_2_ induced AMPK-dependent and pro-survival autophagy in cultured osteoblast cells, which was enhanced by A-769662. Our results suggested that activation of AMPK by H_2_O_2_ is anti-apoptosis and pro-survival in osteoblast cells, probably due to its anti-oxidant, pro-autophagy and ATP preservation abilities, and A-769662-mediated cell-protective effect in osteoblast cells requires AMPK activation. Our study suggests that A-769662 might be further investigated as a novel anti-osteonecrosis agent.

## 1. Introduction

In the pathological condition of osteonecrosis, bones become fragile and will lead to bone fracture if not treated properly. Osteoblasts are important for bone formation and remodeling. Studies have shown that osteonecrosis is associated with oxidative stress in osteoblasts. Elevated reactive oxygen species (ROS) (*i.e.*, hydrogen peroxide (H_2_O_2_)) induce osteoblasts dysfunction and apoptosis, serving as an important contributor of osteonecrosis [1,2]. Different groups have added H_2_O_2_ to cultured osteoblasts to create a cellular model of osteonecrosis [1,2].

Adenosine monophosphate (AMP)-activated protein kinase (AMPK) is a heterotrimer kinase composed of α, β and γ subunits. It is a member of metabolite-sensing kinase family, which plays important roles in metabolic balance [3,4]. Recent studies suggest that AMPK is also important for cell survival under stress conditions. For example, AMPK activation regulates nicotinamide adenine dinucleotide phosphate (NADPH) homeostasis to promote cell survival [5]. Further, AMPK-dependent tuberous sclerosis complex 2 (TSC2) phosphorylation is important for cell survival under energy starvation conditions [6]. Resveratrol, an AMPK activator, improves endurance of mice on a high-calorie diet [7]. Other studies, however, showed that activation of AMPK is somehow pro-apoptotic [8,9,10,11]. AMPK could be activated by H_2_O_2_ [11,12,13]. However, the defensive role of AMPK in H_2_O_2_-induced cell apoptosis is still debatable.

Recent studies have confirmed other functions of AMPK activation. For example, AMPK is important for regulation of autophagy, which is an evolutionarily conserved caspase-independent process of degradation and recycling of long-lived proteins and cytoplasmic-damaged organelles [14,15,16]. Autophagy exerts its pro-survival ability through apoptosis inhibition [14,17]. AMPK promotes autophagy activation through phosphorylation of its downstream target Raptor and consequent inhibition of mammalian target of rapamycin (mTOR) [18,19], as well as through directly activating Ulk1, the autophagy initiator [20,21].

AICAR (5-Aminoimidazole-4-carboxamide-1-β-d-ribofuranoside) is a well-characterized AMPK activator, which becomes *N* 1-(β-d-5'-phosphoribofuranosyl)-5-aminoimidazole-4-carboxamide (ZMP) after taken by the cell to mimic the effect of AMP [22]. A-769662 is another validated AMPK activator that stimulates AMPK via allosteric activation of AMPKα at Thr-172 [23]. A recent study by Meester *et al*., demonstrated that pharmacological activation of AMPK by A-769662 inhibits mesenchymal stem cells (MSCs) proliferation via the regulation of p27 expression [24]; Ducommun *et al.* showed that AMPK activator A-769662 inhibited lipogenesis in primary hepatocytes, co-treatment with AICAR caused profound lipogenesis inhibition through synergistic activation of AMPK [25].

In the current study, we aimed to understand the potential role of AMPK in H_2_O_2_-induced apoptosis of osteoblasts, and to investigative the underlying mechanisms. Here, we mainly focused on the effect of A-769662 on H_2_O_2_-induced osteoblast cell apoptosis.

## 2. Results

### 2.1. A-769662 Inhibits H_2_O_2_-Induced Osteoblast Cell Death

**Figure 1:**
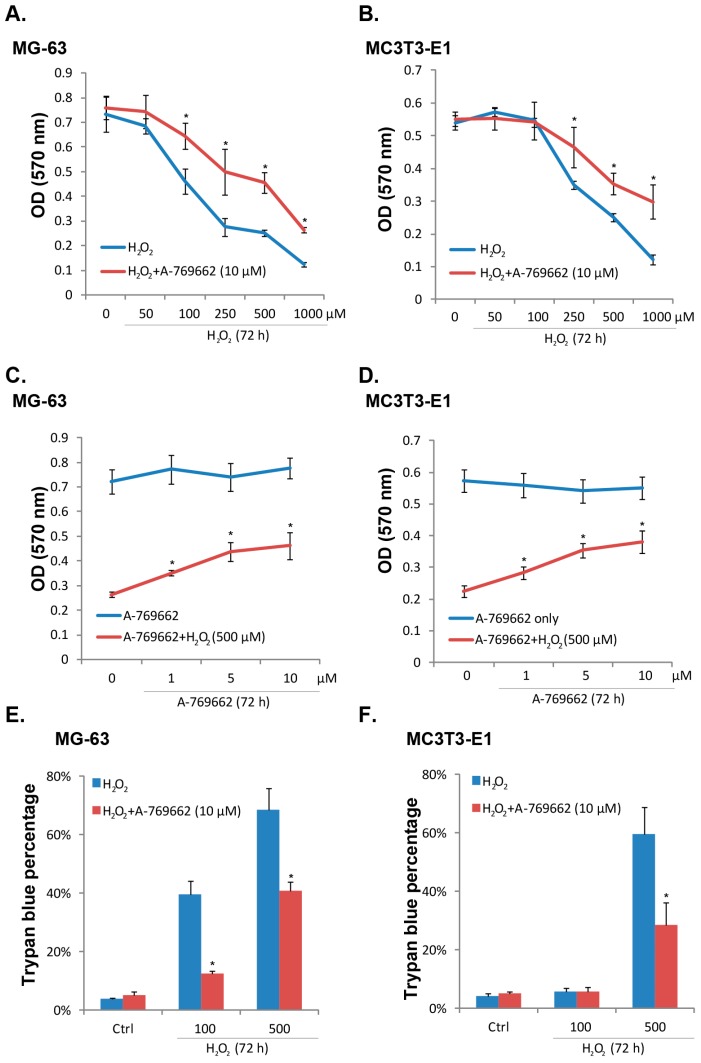
A-769662 inhibits H_2_O_2_-induced osteoblast cell death. Cultured human and mouse osteoblast-like cell lines (MG-63 and MC3T3-E1) were pre-treated with A-769662 (10 μM) for 1 h, followed by hydrogen peroxide (H_2_O_2_, 50–1000 μM) stimulation, cells were further cultured for 72 h, and MTT assay was performed to test cell viability (**A**) and (**B**); trypan blue staining was utilized to test cell death (**E**) and (**F**); MG-63 and MC3T3-E1 cells were pre-treated with different concentration of A-769662 (1, 5 and 10 μM) for 1 h, followed by H_2_O_2_ (500 μM) stimulation, cells were further cultured for 72 h, and 3-[4,5-dimethylthiazol-2-yl]-2,5-diphenyltetrazolium bromide (MTT) assay was performed to test cell viability (**C**) and (**D**). Values in this figure were expressed as mean ± SD, experiments were repeated 3 times and similar results were obtained (same for all figures). * *p* < 0.05 *vs.* group without A-769662.

As shown in Figure 1A,B, hydrogen peroxide (H_2_O_2_, 50–1000 μM) dose-dependently inhibited the viability of human and mouse osteoblast cells (MG-63 and MC3T3-E1 lines). Significantly, co-administration of A-769662 (10 μM) inhibited viability loss by H_2_O_2_ in both cell lines (Figure 1A,B). Meanwhile, the effect of A-769662 was dose-dependent (Figure 1C,D), and A-769662 at 10 μM showed best cell-protective effect (Figure 1C,D). Meanwhile, as demonstrated in Figure 1E,F, H_2_O_2_-induced death of osteoblasts, shown by the increased number of trypan blue stained cells, was also inhibited by A-769662 (10 μM). Thus, A-769662 inhibits H_2_O_2_-induced osteoblast cell death.

### 2.2. A-769662 Suppresses H_2_O_2_-Induced Osteoblast Cell Apoptosis

**Figure 2:**
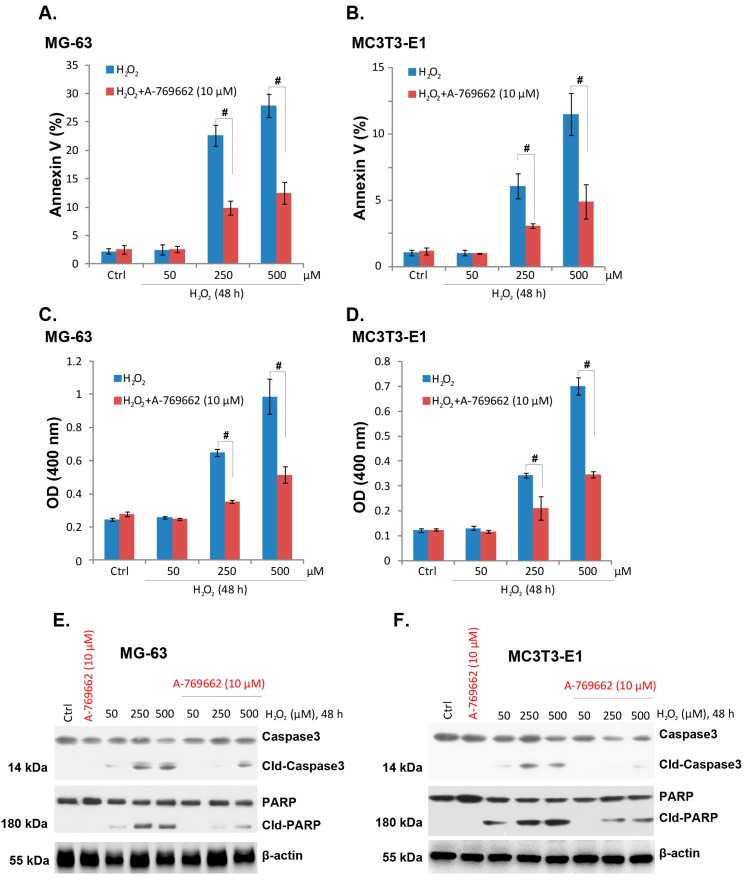
A-769662 suppresses H_2_O_2_-induced osteoblast cell apoptosis. MG-63 and MC3T3-E1 cells were pre-treated with A-769662 (10 μM) for 1 h, followed by hydrogen peroxide (H_2_O_2_, 50–500 μM) stimulation, cells were further cultured for 48 h, and Annexin V fluorescence-activated cell sorting (FACS) assay (**A**) and (**B**) and histone-DNA ELISA assay (**C**) and (**D**) were performed to test cell apoptosis; the expressions of PARP (cleaved and regular), caspase-3 (cleaved and regular) and β-actin were tested by western blots (**E**) and (**F**). # *p* < 0.05.

Results in Figure 1 showed that A-769662 suppressed H_2_O_2_-induced osteoblast cell death. Next, we tested whether A-769662 affected osteoblast cell apoptosis. As mentioned, cell apoptosis was tested by histone-DNA apoptosis enzyme linked immunosorbent assay (ELISA) assay and Annexin V FACS assay. Results in Figure 2A–D demonstrated that H_2_O_2_ (50–500 μM) induced cell apoptosis in both lines, as the histone DNA ELISA optical density (OD) and the percentage of Annexin V cells increased significantly after H_2_O_2_ treatment (Figure 2A–D). Notably, co-administration with A-769662 (10 μM) dramatically suppressed H_2_O_2_-induced cell apoptosis in both cell lines (Figure 2A,D). H_2_O_2_-induced cleavage of PARP and caspase-3 was also inhibited by A-769662 (Figure 2E,F). These results indicated that A-769662 significantly suppressed H_2_O_2_-induced osteoblast cell apoptosis.

### 2.3. A-769662-Induced Pro-Survival Effect against H_2_O_2_ Requires AMPK Activation

**Figure 3:**
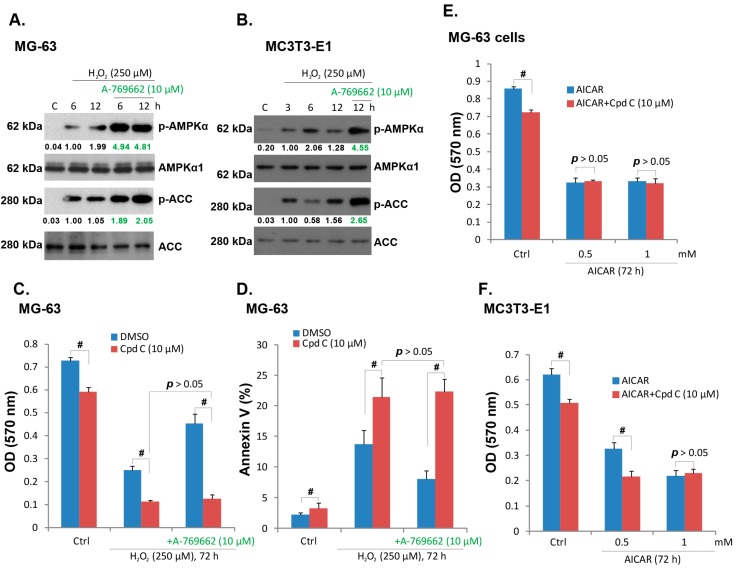
A-769662-induced pro-survival effect against H_2_O_2_ requires AMP-activated protein kinase (AMPK) activation. MG-63 and MC3T3-E1 cells were pre-treated with A-769662 (10 μM) for 1 h, followed by hydrogen peroxide (H_2_O_2_, 250 μM) stimulation, cells were further cultured for indicated time points, and western blots were performed to test phospho(p)- and total (t)-AMPKα, p- and t- ACC (**A**) and (**B**); MG-63 and MC3T3-E1 cells were pre-treated with AMPK inhibitor compound C (Cpd C, 10 μM) for 2 h, followed by hydrogen peroxide (H_2_O_2_, 250 μM) or H_2_O_2_+ A-769662 (10 μM) stimulation, cells were further cultured, cell viability (**C**) and Annexin V percentage (**D**) were tested as described; MG-63 and MC3T3-E1 cells were pre-treated with compound C (Cpd C, 10 μM) for 2 h, followed by AICAR (0.5 and 1 mM) stimulation, cells were further cultured for 72 h when cell viability was examined (**E**) and (**F**). # *p* < 0.05.

As discussed, A-769662 is a well-known AMPK agonist [26]. Thus we tested the involvement of AMPK in A-769662-induced protective effect against H_2_O_2_. Western blot results in Figure 3A,B showed that H_2_O_2_ induced a moderate AMPK activation, and the expressions of phospho (p)-AMPKα and p-acetyl CoA carboxylase (ACC) increased after H_2_O_2_ stimulation in both MG-63 and MC3T3-E1 cells. Notably, co-administration with A-769662 (10 μM) dramatically enhanced AMPK activation (AMPKα/ACC phosphorylation) by H_2_O_2_ (Figure 3A,B). Compound C (Cpd C), the AMPK inhibitor, aggravated H_2_O_2_-induced MG-63 cell damage, with increased cell viability loss and Annexin V positive cells observed (Figure 3C,D). Importantly, A-769662-induced protective effect against H_2_O_2_ was abolished by compound C (Figure 3C,D). These results indicated that A-769662-induced protective effect in osteoblast cells requires AMPK activation. Note that compound C alone also slightly inhibited MG-63 cell viability (Figure 3C,D), indicating that basal AMPK activation might be important for cell survival. Interestingly, 5-aminoimidazole-4-carboxamide-1-β-d-ribofuranoside (AICAR), another well-known AMPK agonist [27], dramatically inhibited viability of MG-63 and MC3T3-E1 cells alone (Figure 3E,F). Such an effect of AICAR appeared not dependent on AMPK activation, as compound C failed to rescue it (Figure 3E,F). These results indicate that AICAR induces AMPK-independent osteoblast cell death, a phenomenon that is seen in other cell lines [28].

### 2.4. A-769662 Alleviates Reactive Oxygen Species (ROS) Accumulation and ATP Depletion Caused by H_2_O_2_

**Figure 4:**
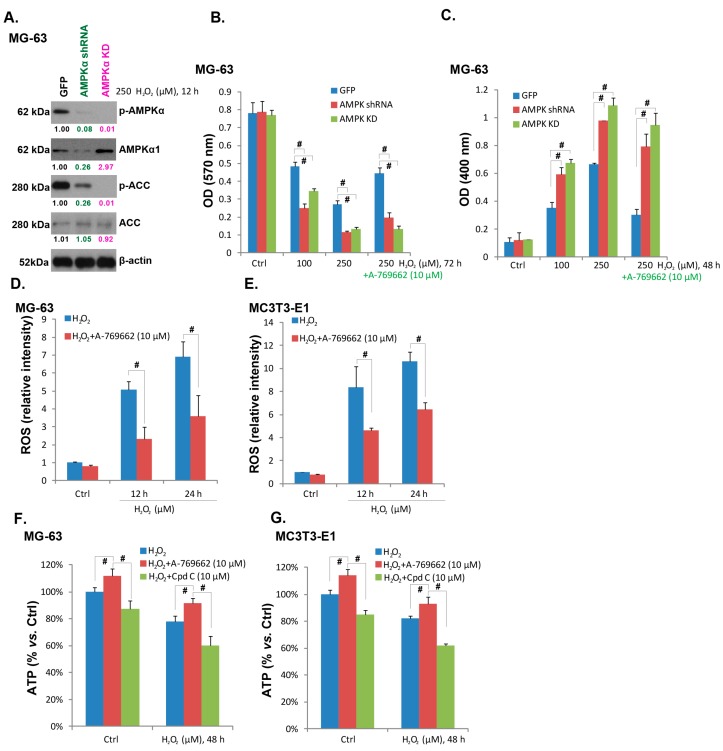
A-769662 alleviates ROS accumulation and ATP depletion caused by H_2_O_2_. The expressions of AMPKα (p- and t-), ACC (p- and t-) and β-actin in hydrogen peroxide (H_2_O_2_, 250 μM) stimulated MG-63 cells transfected with GFP-shRNA, AMPKα shRNA lentivirus or AMPKα kinase dead (KD) plasmid (**A**); Above cells were also subjected to H_2_O_2_ stimulation (100 and 250 μM) in the presence or absence of A-769662 (10 μM), cell viability and apoptosis were analyzed by MTT assay (**B**) and histone-DNA apoptosis ELISA assay respectively (**C**); The effect of A-769662 (10 μM, 1 h pretreatment) on H_2_O_2_-induced ROS accumulation was tested as described (**D**) and (**E**); The effect of A-769662 (10 μM, 1 h pretreatment) or compound C (Cpd C, 10 μM, 1 h pretreatment) on cellular ATP content in H_2_O_2_-stimulated MG-63 and MC3T3-E1 cells were analyzed (**F**) and (**G**). # *p* < 0.05.

To further confirm that AMPK activation is required for A-769662-induced pro-survival effect, we generated stable MG-63 cell lines expressing AMPKα shRNA (AMPKα knockdown cell line) or kinase dead (KD) AMPKα (T172A, AMPKα KD cell line). As shown in Figure 4A, AMPKα stable knockdown or KD mutation almost blocked AMPK activation by H_2_O_2_. H_2_O_2_-induced MG-63 cell viability loss (Figure 4B) and apoptosis (Figure 4C) were exacerbated in AMPKα-knockdown or mutated cells, confirming that activation of AMPK by H_2_O_2_ is a pro-survival signaling in osteoblasts. Meanwhile, A-769662-induced pro-survival effect against H_2_O_2_ was almost blocked with AMPKα knockdown or mutation (Figure 4B,C), further supporting the involvement of AMPK activation in the process. A recent study by Jeon *et al.* showed the antioxidant ability of AMPK [5], and AMPK activation suppresses oxidative stress through inhibiting NADPH depletion and promoting NADPH synthesis [5]. As shown in Figure 4D,E, A-769662 alleviated ROS accumulation in H_2_O_2_-treated osteoblasts, further confirming its anti-oxidant ability. Meanwhile, H_2_O_2_ stimulation decreased cellular ATP content in both cell lines (Figure 4E,F), which was partly recued by A-769662, but was exacerbated by Compound C (Figure 4E,F). These results suggested that A-769662 alleviates ROS accumulation and ATP depletion caused by H_2_O_2_, which may serve as the main mechanisms of its cell-protective efficiency.

### 2.5. A-769662 Facilitates H_2_O_2_-Induced Autophagy Activation

**Figure 5:**
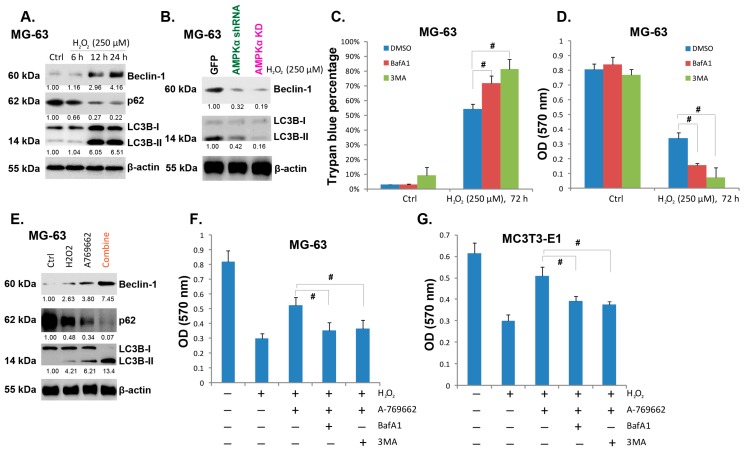
A-769662 facilitates H_2_O_2_-induced autophagy activation. MG-63 cells were treated with hydrogen peroxide (H_2_O_2_, 250 μM), cells were further cultured for indicated time points, and western blots were performed to test LC3B, Beclin-1, p62 and β-actin (**A**); The expressions of LC3B, Beclin-1, p62 and β-actin in H_2_O_2_ (250 μM) stimulated MG-63 cells transfected with GFP-shRNA, AMPKα shRNA lentivirus or AMPKα kinase dead (KD) plasmid were tested (**B**); MG-63 cells were pre-treated with 3-methyladenine (3-MA, 1 mM), or Bafilomycin A1 (BafA1, 0.1 μM) for 1 h, followed by H_2_O_2_ (250 μM) stimulation for 72 h, cell viability (**C**) and trypan blue assay (**D**) were performed; MG-63 cells were treated with H_2_O_2_ (250 μM), A-769662 (10 μM) or both for 24 h, the expressions of LC3B, Beclin-1, p62 and β-actin were tested (**E**); The viability of MG-63 or MC3T3-E1 cells with indicated treatment for 72 h was tested by MTT assay (**F** and **G**). # *p* < 0.05.

As discussed, one main consequence of AMPK activation is autophagy, which is also cyto-protective. Here, we found that H_2_O_2_ activated autophagy is MG-63 cells, which was demonstrated by p62 degradation, LC3-II production and Beclin-1 expression (Figure 5A). AMPK activation was required for H_2_O_2_-induced autophagy induction in MG-63 cells, and AMPK shRNA depletion or kinase-dead (KD) mutation inhibited autophagy activation by H_2_O_2_ (Figure 5B). H_2_O_2_-induced autophagy appeared cytoprotective, as inhibition of autophagy by two inhibitors (3-MA and BafA1) exaggerated H_2_O_2_-induced viability loss and cell death (Figure 5C,D). On the other hand, the AMPK activator A-769662 facilitated H_2_O_2_-induced autophagy activation (Figure 5E), while A-769662-induced pro-survival effect against H_2_O_2_ was alleviated by the autophagy inhibitors in both MG-63 and MC3T3-E1 cells (Figure 5F,G). Together, these data suggest that H_2_O_2_ induces AMPK-dependent and cyto-protective autophagy in cultured osteoblasts. A-769662-induced pro-survival effect against H_2_O_2_ is mediated, at least in part, by its ability to activate autophagy (Figure 5).

## 3. Discussion

Here we reported that A-769662 inhibited H_2_O_2_-induced death and apoptosis in both human and mouse osteoblast cells. AMPK inhibition by its inhibitor compound C, by targeted shRNA-mediated silencing or kinase dead (KD) mutation exacerbated H_2_O_2_-induced death and apoptosis of osteoblast cells. A-769662-induced cell-protective effect was diminished when AMPK was inactivated or depleted. We found that A-769662 inhibited ROS accumulation and ATP depletion by H_2_O_2_, which might mediate its pro-survival effect. Further, H_2_O_2_ induced AMPK-dependent and cyto-protective autophagy in cultured osteoblast cells, which was enhanced by A-769662. Based on these results, we suggested that activation of AMPK by H_2_O_2_ might serve as a negative regulator to promote osteoblast cell survival.

Recent studies have shed lights on how AMPK mediates cell survival. AMPK activation attenuates oxidative stress-mediated cell death through promoting NADPH synthesis and limiting its consumption [5]. Thus, in addition to its function in ATP homeostasis, the AMPK/acetyl-CoA carboxylase (ACC) signaling axis works as an anti-oxidant to maintain NADPH homeostasis, and to decrease ROS accumulation, rescuing cells from oxidative stresses [5]. In the current study, we found that H_2_O_2_-induced oxidative stress in osteoblast cells was inhibited A-769662, but was aggravated by compound C, suggesting that AMPK activation and A-769662 might have the similar anti-oxidant effect.

In almost all eukaryotic cells, AMPK constitutes a molecular hub for cellular metabolic control. When cells facing metabolic stress (*i.e.*, ischemia/hypoxia and glucose deprivation), AMP/ATP ratio will increase to activate AMPK [6]. TSC2 and its phosphorylation by AMPK protect cells from energy deprivation-induced apoptosis [6]. In the current study, we also observed that ATP level was decreased when osteoblast cells stimulated with H_2_O_2_, which might be the key mechanism to activate AMPK. Activated AMPK then tried to maintain ATP level and recue cells. Forced activation of AMPK by A-769662 could then increase cellular ATP level and to maintain cell survival.

Autophagy begins with formation of double membrane vesicles (also known as autophagosomes) in the cytoplasm, which will “eat” cytoplasmic materials and fuse with lysosome, where the contents will be “recycled” by acidic lysosomal hydrolases. In this process, LC3B-I is cleaved and converted to LC3B-II, which is associated with Beclin-1 and other components to form pre-autophagosomal structures. In the current study, we observed LC3B-II and Beclin-1 increase in osteoblast cells with H_2_O_2_ and/or A769662 stimulation, indicating autophagy activation. The fact that autophagy inhibitors (3-MA and BafA1) enhanced H_2_O_2_-induced cytotoxicity suggests that autophagy activation is cyto-protective in osteoblast cells, and A-769662-mediated pro-survival effect could be associated with its ability to promote autophagy activation.

## 4. Materials and Methods

### 4.1. Chemicals and Regents

A-769662 was purchased from Cayman Chemical (Ann Arbor, MI, USA). Compound C and AICAR were obtained from Calbiochem (Darmstadt, Germany). 3-methyladenine (3-MA), Bafilomycin A1 (BafA1) and H_2_O_2_ were purchased from Sigma (Shanghai, China).

### 4.2. Antibodies

Anti-AMPKα1, acetyl-CoA carboxylase (ACC), rabbit and mouse horseradish peroxidase (HRP)-conjugated IgG antibodies were purchased from Santa Cruz Biotechnology (Santa Cruz, CA, USA). Antibodies against phospho(p)-AMPKα (Thr 172), p-ACC (Ser 79), light chain 3 B (LC3B), Beclin-1, p62 and β-actin were purchased form Cell Signaling Tech. (Denver, MA, USA).

### 4.3. Cell Culture

Human osteoblast-like cell line MG63 and mouse osteoblast cell line MC3T3-E1 were grown in Dulbecco’s modified Eagle’s medium-high glucose (DMEM) and α-MEM, respectively, supplemented with 10% fetal bovine serum (FBS), 50 units/mL penicillin and 50 μg per streptomycin in a humidified atmosphere of 5% CO_2_. Cultures were trypsinized upon confluence and sub-cultured into 12-, 6-, or 96-well plates for further experiments.

### 4.4. Cell Viability Assay

Methyl thiazolyl tetrazolium (MTT) assays were performed to assess the cell viability. Briefly, MC3T3-E1 and MG63 cells were planted into 96-well plates. After incubation overnight, the medium was replaced with fresh medium with or without H_2_O_2_ at indicated concentrations for various time points. Some of wells were also pre-treated with indicated inhibitors. Six wells were included in each concentration. At the end of each treatment, 20 μL of MTT (5 mg/mL, Sigma) was added for 4 h. Then the medium was discarded carefully and 150 μL of DMSO was added. Absorbance was recorded at 570 nm with the Universal Microplate Reader (Bio-Tek instruments, Winooski, VT, USA ) using wells without cells as blanks.

### 4.5. Western Blot and Data Analysis

After treatment, cells were lysed immediately in the lysis buffer (62.5 mM Tris-HCl, pH 6.8, 2% SDS, 10% glycerol, 50 mM dithiothreitol, 0.01% bromophenol blue) for 5 min at 95 °C. Cell lysates were analyzed by SDS/PAGE and transferred electrophoretically to poly-vinylidene fluoride (PVDF) membranes (Bio-Rad Corp, Hercules, CA, USA). Blots were probed with specific antibodies and immuno-reactive proteins were revealed by the enhanced chemiluminescence (ECL) kit (Santa Cruz Biotechnology Inc., Santa Cruz, CA, USA). The band intensity of each blot was quantified by Image J software before normalizing with the corresponding loading controls, and the value was expressed as fold or percentage change *vs.* the band labeled “1.00”.

### 4.6. Cell Apoptosis Assay by Annexin V Staining

After treatment, the apoptosis of osteoblasts was detected by the Annexin V fluorescence-activated cell sorting (FACS). Briefly, one million cells with indicated treatments were stained with FITC-Annexin V and propidium iodide (PI) (Beyotime, Shanghai, China). Both early (annexin V^+^/PI^−^) and late (annexin V^+^/PI^+^) apoptotic cells were sorted by the fluorescence activated cell sorter (FACS) machine (Becton Dickinson FACS Calibur, San Jose, CA, USA). The percentage of apoptotic cells was recorded.

### 4.7. Cell Apoptosis Assay by Histone-DNA Enzyme-Linked Immunosorbent Assay (ELISA) PLUS Kit

Osteoblasts’ apoptosis was also quantified by histone-DNA ELISA PLUS (Roche Applied Science, Shanghai, China) according to the manufacturer’s protocol. Briefly, the cytoplasmic histone/DNA fragments from cells were extracted and bound to the immobilized anti-histone antibody. Subsequently, the peroxidase-conjugated anti-DNA antibody was added for the detection of immobilized histone/DNA fragments. After addition of substrate for peroxidase, the spectrophotometric absorbance of the samples was determined using a plate reader at a test wavelength of 400 nm.

### 4.8. ROS Assay

Cellular ROS level was measured by a DCFH-DA fluorescent dye (Molecular Probes/Invitrogen Eugene, OR, USA). After treatment, the osteoblasts were incubated with 10 μM of DCFH-DA at 37 °C for 20 min and then washed twice with PBS. The cells were then analyzed for fluorescence using the flow cytometer. Cellular ROS level in the treatment group was normalized to that of the control group.

### 4.9. Trypan Blue Staining of “Dead” Cells

The number of dead cells (trypan blue positive) after indicated treatment was counted, and the percentage (%) of “dead” cells was calculated by the number of the trypan blue positive cells divided by the total number of the cells.

### 4.10. Stable AMPKα Knockdown through Lentiviral Infection

MG-63 cells were seeded in a six-well plate in growth medium. The lentiviral particles containing human AMPKα1/2 shRNA (10 μL/mL, Santa Cruz Biotech, Santa Cruz, CA, USA) were added to the cells, after 12 h, the lentiviral particles containing medium was replaced by fresh growth medium, and cells were further cultured for another 24 h. Puromycin (1 μg/mL) was added to select resistant stable colonies. The expression of AMPKα in stable cells was detected by western blots. Control cells were infected with GFP-shRNA containing lentiviral particles (Kaiji Biotech, Nanjing, China).

### 4.11. AMPK Kinase Dead (KD) Mutation and Stable Cell Selection

As previously reported [29], the human AMPK-α1 cDNA was amplified from MG-63 cell cDNA, and was sub-cloned into the BamHI site of pcDNA3.1 (Invitrogen, Shanghai, China). The kinase dead (KD) mutant of AMPK-α1 (AMPK-α1-T172A) was created by mutating the Thr172 residue into Ala as previously reported [30,31]. KD-AMPK-α1 cDNA was transfected to the MG-63 cells through Lipofectamine 2000 protocol [29], stable cells were selected through neomycin (1 μg/mL) as described.

### 4.12. Measurement of Intracellular ATP Content

As reported [32], after treatment, the intracellular ATP content in osteoblasts was measured using the Molecular Probes’ ATP Determination Kit (Kaiji, Nanjing, China) according to the manufacturer’s directions [32]. This kit is based on the bioluminescence detection of ATP, using recombinant firefly luciferase and its substrate, luciferin. Total chemiluminescence was collected by a luminometer. The amount of ATP from tested cells was quantified by comparison to a calibration curve using ATP as the standard. The value in the treatment group was normalized to that of control group. All experiments were performed in triplicate.

### 4.13. Statistical Analysis

Statistical analyses were performed by analysis of variance (ANOVA), and *p* < 0.05 was considered statistically significant.

## 5. Conclusions

In conclusion, our study indicated that A-769662 significantly protects against H_2_O_2_-induced apoptosis of osteoblast cells. A-769662 might be further investigated as a novel anti-osteonecrosis agent.
